# Bispecific antibody targeting CD40 and HER2 potentiates therapeutic efficacy by reprogramming macrophages within the tumour microenvironment

**DOI:** 10.1002/ctm2.70428

**Published:** 2025-07-29

**Authors:** Na Li, Ruonan Li, Qiongqiong Ma, Xiaoqi Zhang, Wenxuan Ma, Yi Wang, Baoxin Duan, Kailu Yang, Dongping Zhang, Jiashuo Zhang, Manping Gu, Yaxing Wu, Jiajin Sun, Huawei Wang, Anqi Li, Fuquan Chen, Yiyang Bai, Yujie Tian, Xin Li, Yingbin Yan, Wei Wang, Hongkai Zhang, Yuan Wang

**Affiliations:** ^1^ Institutes of Biomedical Sciences, School of Life Sciences Inner Mongolia University Hohhot China; ^2^ State Key Laboratory of Medicinal Chemical Biology and College of Life Sciences Nankai University Tianjin China; ^3^ Department of Oromaxillofacial—Head and Neck Surgery, Tianjin Stomatological Hospital The Affiliated Stomatological Hospital of Nankai University Tianjin China; ^4^ Shanghai Institute for Advanced Immunochemical Studies ShanghaiTech University Shanghai China

1

Dear Editor,

CD40, a stimulatory receptor that is highly expressed primarily on antigen‐presenting cells (APCs) plays a pivotal function in mediating immune system activation.^1^, ^2^ Although agonistic CD40 antibodies have demonstrated some efficacy in early‐phase clinical trials, they have been hampered by both dependency of FcγR‐mediated crosslinking and systemic toxicity.^3^, ^4^ TAA‐CD40 bispecific antibodies (BsAbs) represent a promising strategy to overcome these limitations,^5^, ^6^ but their in vivo therapeutic mechanisms remain poorly understood. In this study, we developed a CD40‒HER2 BsAb that demonstrated potent antitumour efficacy while evading the toxicity limitations commonly associated with CD40 agonists. Mechanistically, CD40‒HER2 BsAb treatment primarily reprogrammed macrophages to boost the immune response in vivo.

To achieve tumour‐localised CD40 stimulation without systemic FcγR crosslinking, we designed CD40‒HER2 BsAbs with an N297A Fc mutation, which eliminates FcγR binding to prevent antibody‐dependent cellular cytotoxicity against CD40‐positive APCs and HER2‐independent CD40 activation (Figure 1A).^7^, ^8^, ^9^ While BsAb‐1 and BsAb‐5 exhibited low production yields (Table S1). BsAb‐2 to BsAb‐4 derived from trastuzumab and BsAb7 with a HER2‐binding Fc mutation showed the capacity to target CD40 and HER2 (Figure 1B). Further Jurkat/NF‐κB‐GFP‐hCD40 reporter cells assay showed that BsAb‐7 specifically activated reporter cells with maximal intensity in the presence of HER2‐positive CHO‒HER2 cells (Figure 1C,D).

**FIGURE 1:**
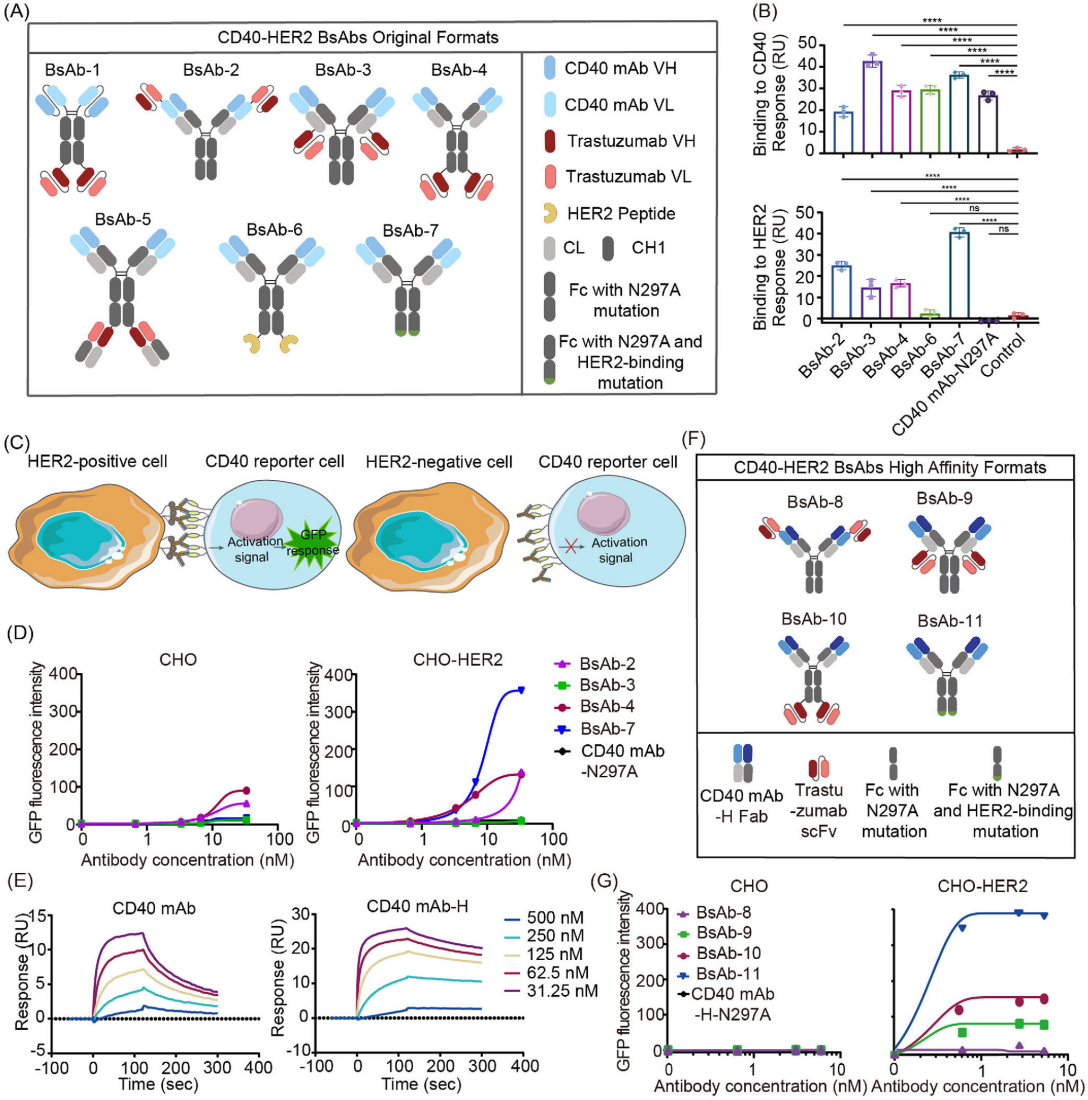
Generation and optimization of CD40‒HER2 bispecific antibodies (BsAbs). (A) Schematic diagram of different formats of CD40‒HER2 BsAbs. (B) The binding of CD40‒HER2 BsAbs to both CD40 (up) and HER2 (down) was assessed by surface plasmon resonance. (C) Schematic of the CD40 activation reporter cell assay. CD40 activation upon HER2‐mediated BsAb crosslinking induces GFP expression in reporter cells. (D) CD40 activation in reporter cells stimulated by different formats of BsAbs in the presence of CHO‒HER2 or CHO cells. (E) The affinity of CD40 mAb and CD40 mAb‐H for CD40 was measured by surface plasmon resonance. (F) Schematic diagram of CD40‒HER2 BsAbs with high affinity for CD40. (G) CD40 activation in reporter cells stimulated by different formats of BsAbs in the presence of CHO‒HER2 or CHO cells. ^*^ *p *< .05, ^**^ *p* < .01, ^***^ *p* < .001, ^****^ *p* < .0001.

We next investigated the impact of affinity and epitope on CD40‒HER2 BsAb activities. Affinity maturation significantly enhanced the binding affinity and agonistic activity of CD40 mAb‐H compared to its parental CD40 mAb (Figures 1E and S1A). Four formats of CD40‒HER2 BsAbs with high CD40 affinity demonstrated binding to both CD40 and HER2 (Figures 1F and S1B). Increased affinity enhanced the activity of the CD40‒HER2 BsAbs, and the Fc mutation with HER2‐binding ability format (BsAb‐11) had the highest agonistic capacity (Figure 1G). However, after epitope exchange with APX005M and pertuzumab (Figure S1C‒E),^10^ APX005M‐derived BsAb‐15 and pertuzumab‐derived BsAb‐12 to BsAb‐14 did not further enhance the agonistic activity compared to CD40 mAb‐H‐derived BsAb‐11 (Figure S1F). Additionally, CD40‒HER2 BsAb‐11 significantly activated the reporter cells upon incubation with HER2‐high‐expressing SKBR3 and BT474 cells, but not with HER2‐low‐expressing T47D and MDA‐MB‐231 cells (Figure S1G,H). These results indicating that affinity is an important variable to take into account in the development of BsAb.

CD40‒HER2 BsAb‐11 exhibited binding affinities (*K*_D_) of 37.5 nM for CD40 and 167.1 nM for HER2, with EC50 values of 2 and 30 nM for cell surface binding, respectively (Figure S2A,B). It significantly enhanced cell‐to‐cell interactions and improved the uptake efficiency of mature dendritic cell (DCs) on HER2‐coated fluorescent spheroids (Figures 2A‒C and S2C,D). Furthermore, CD40‒HER2 BsAb‐11 delayed tumour growth in both MC38‒hHER2 and MB49‒hHER2 hCD40tg mouse models compared to CD40 mAb‐H‐N297A (Figure S2E,F). It effectively increased tumour‐infiltrating CD3+, CD4+ and CD8+ T cells while decreasing Tregs (Figures 2D‒F and S3). However, it had a limited effect on the proportion of DCs in tumour tissue, in contrast to CD40 mAb‐H‐mIgG1, which markedly decreased DCs (Figure 2G). Additionally, CD40‒HER2 BsAb‐11 showed limited liver toxicity, with stable serum AST/ALT levels, preserved liver immune cell proportions, and no histopathological damage in liver and kidney tissues, in contrast to CD40 mAb‐H‐mIgG1 (Figures 2H,I and S2G). These results highlight CD40‒HER2 BsAb‐11 as a potent immune activator with dual targeting and reduced toxicity compared to conventional CD40 agonists.

**FIGURE 2:**
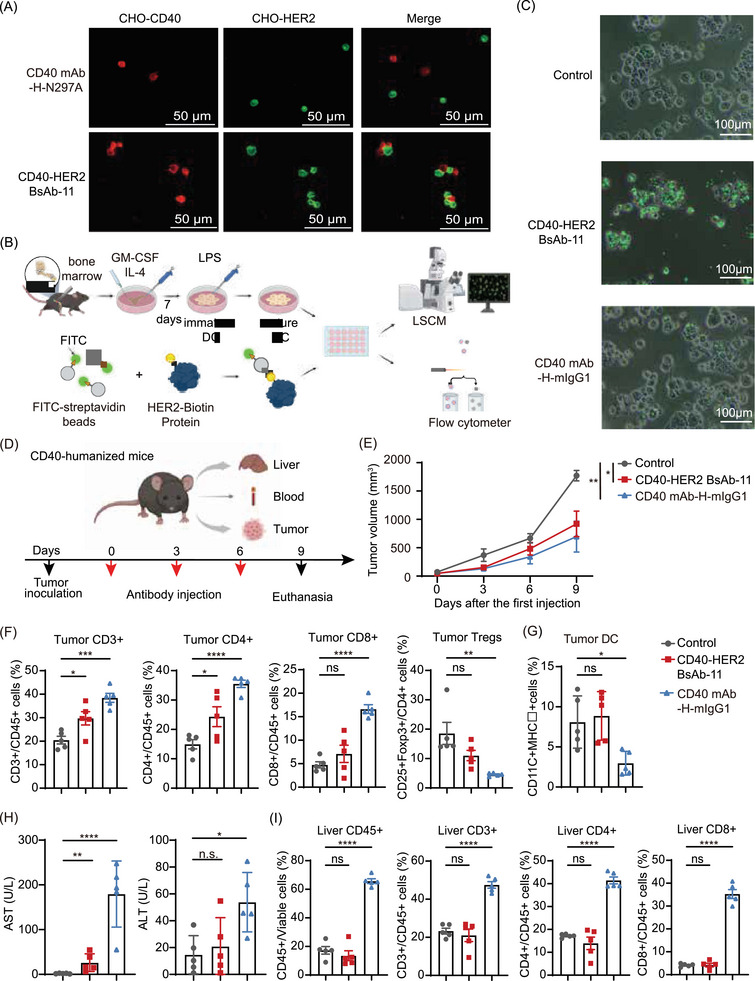
In vitro functionality and in vivo activity of CD40‒HER2 bispecific antibodies (BsAbs). (A) Simultaneous binding of CD40‒HER2 BsAb‐11 to HER2‐expressing cells (green) and CD40‐expressing cells (red), visualised by confocal microscopy. (B) Schematic diagram of in vitro bone marrow‐derived dendritic cell (BMDC) induction and phagocytosis of HER2‐positive beads by DCs. (C) Phagocytosis of HER2+ beads by BMDC in the presence or absence of CD40‒HER2 BsAb‐11 or CD40 mAb‐H‐mIgG1 was detected by fluorescence microscopy. (D) The experimental treatment design for MB49‒hHER2 tumour‐bearing hCD40tg mice. (E) Tumour volume was measured at indicated times and plotted as mean ± SEM (standard error of the mean) (*n* = 5). (F and G) Flow cytometric analysis of T cells (F) and DCs (G) from tumour of MB49‐hHER2‐bearing mice treated as indicated (*n* = 5). (H) Blood was collected from MB49‐hHER2‐bearing hCD40tg mice treated as indicated (*n* = 5), hepatorenal toxicity indexes aspartate aminotransferase (AST) and alanine aminotransferase (ALT) were measured. Data are represented as mean ± SEM. (I) Flow cytometric analysis of T cells from liver of MB49‐hHER2‐bearing mice treated as indicated (*n* = 5). ^*^ *p *< .05, ^**^ *p* < .01, ^***^ *p* < .001, ^****^ *p* < .0001.

Single‐cell RNA sequencing analysis of 29 347 tumour‐infiltrating immune cells in the MB49‒hHER2 model identified 13 major clusters (Figures 3A and S4A and Table S2). Three DC subsets were characterised, with CD40‒HER2 BsAb‐11 showing minimal effects on their proportions and functions compared to CD40 mAb‐H‐mIgG1, suggesting that dual targeting of BsAb may hinder DC migration (Figures S4A and 3B,C). CD40‒HER2 BsAb‐11 treatment upregulated antigen‐presenting molecules (*Cd74*, *H2‐dmb2*) and downregulated *PD‐L1/Cd274* in B cells (Figure S4B). Macrophage profiling revealed that CD40‒HER2 BsAb‐11 induced M1‐like polarisation (*Cxcl9*) while reducing M2 markers (*Mrc1*, *Spp1*) and oxidative phosphorylation, driving a pro‐inflammatory phenotype shift (Figures 3D,E and S4C‒E). T/NK cells were classified into 14 subclusters, with BsAb‐11 enhancing cytotoxic, exhausted and proliferative CD8+ T‐cell subsets along with Th1‐like CD4+ T cells, while suppressing Tregs (Figures 3F,G and S4F). Overall, CD40‒HER2 BsAb‐11 primarily enhances macrophage and B‐cell responses rather than DCs, activates T‐cell responses, and synergistically boosts antitumour immunity.

**FIGURE 3:**
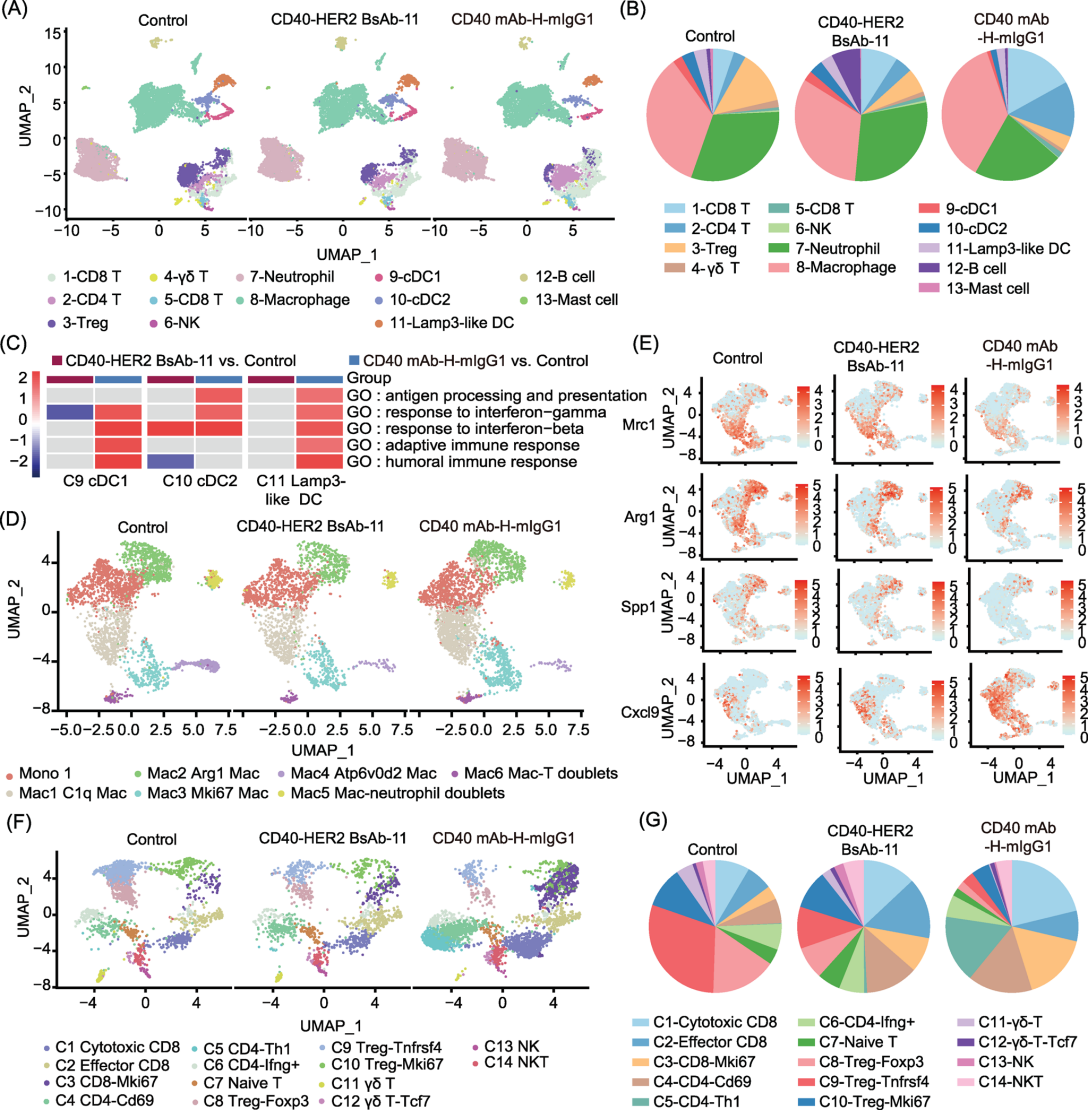
Analysis of tumour‐infiltrating immune cells by single‐cell RNA sequencing (scRNA‐seq) in MB49‒hHER2 model. (A) The CD45+ tumour‐infiltrating immune cells from control, CD40‒HER2 BsAb‐11 and CD40 mAb‐H‐mIgG1 treated mice were isolated and prepared for sequencing. The immune cells were clustered and represented as Uniform manifold approximation and projection (UMAP) plot. (B) Distribution of immune cell clusters in different treatment groups. (C) Heatmap of the Gene Ontology (GO) signalling pathway analysis of CD40‒HER2 BsAb‐11 versus control and CD40 mAb‐H‐mIgG1 versus control in DC clusters. (D) UMAP showing monocyte reclusters analysed by scRNA‐seq. (E) Expression of selected genes in macrophages between treatment groups. (F) UMAP showing T‐cell reclusters analysed by scRNA‐seq. (G) Distribution of immune cell clusters in all T cells.

In vivo depletion assays confirmed that CD40‒HER2 BsAb‐11 inhibited tumour growth mainly through macrophages, B cells and T cells, as their depletion restored tumour growth (Figures 4A,B and S5A,B). In contrast, CD40 mAb‐H‐mIgG1 treatment showed no significant tumour growth difference upon macrophage depletion, highlighting distinct mechanisms between BsAb‐11 and CD40 agonist (Figure 4C). CD40‒HER2 BsAb‐11 also increased tumour‐infiltrating granzyme B+ CD8+ T cells and iNOS+ macrophages (Figure 4D). In vitro, it dose dependently enhanced MHC II‒OVA complex formation and CD86 expression in macrophages co‐cultured with HER2+/OVA+ MC38 cells, indicating dual activation of antigen presentation and costimulatory pathways (Figure 4E,F). Additionally, CD40‒HER2 BsAb‐11 activated CD86 expression in human B cells co‐cultured with HER2+ SKBR3 cells, whereas CD40 mAb‐H‐N297A had minimal effect (Figure 4G).

**FIGURE 4:**
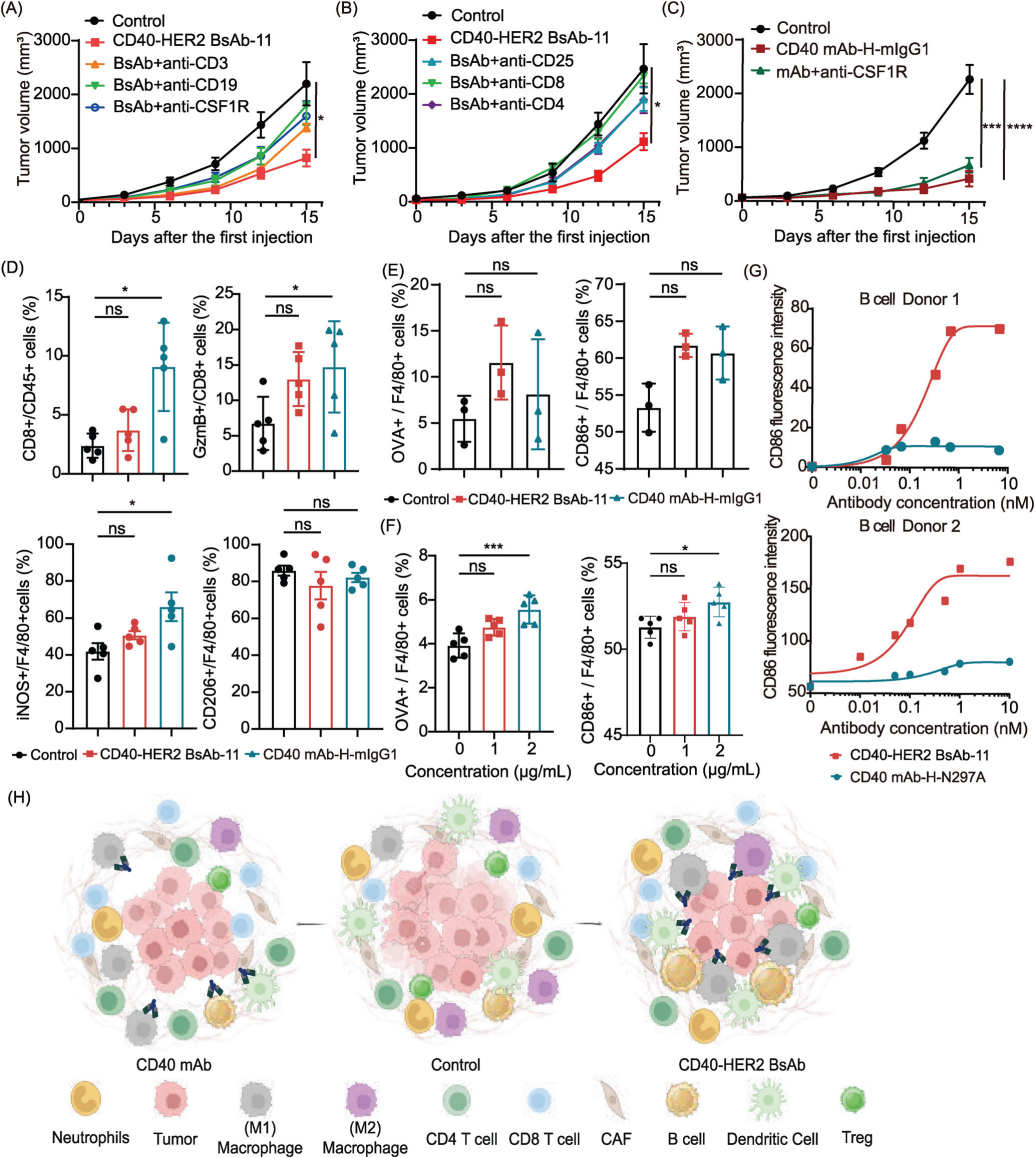
CD40‒HER2 BsAb‐11 exerted antitumour effects through macrophages and B cells. (A and B) Tumour growth in MB49‒hHER2‐bearing hCD40tg mice following immune cell depletion and CD40‒HER2 BsAb‐11 treatment. Depleted populations: macrophages, B cells, T cells (*n* = 6) (A); CD4+ T cells, CD8+ T cells, Tregs (*n* = 5) (B). (C) Tumour growth in macrophage‐depleted mice treated with CD40 mAb‐H‐mIgG1. (D) Flow cytometric analysis of CD8+ T cells and macrophages from tumour of MB49‐hHER2‐bearing mice treated as indicated (*n* = 5). (E and F) Peritoneal macrophages from hCD40tg mice were co‐cultured with MC38‒hHER2‒OVA cells and treated with different antibodies (E) or different concentrations CD40‒HER2 BsAb‐11 (F), the activation of macrophages and presentation of OVA were assessed using flow cytometry. (G) CD86 activation marker expression on human B cells stimulated by CD40‒HER2 BsAb‐11 or CD40 mAb‐H‐N297A with SKBR3 cells. (H) CD40‒HER2 BsAb therapy potentiated the antitumour efficacy by remodelling macrophages and activating B cells, along with increasing the proportion of CD8+ and CD4+ T cells and decreasing the number of Tregs. ^*^ *p *< .05, ^**^ *p* < .01, ^***^ *p* < .001, ^****^ *p* < .0001.

In conclusion, our data demonstrated that the HER2‐targeting CD40 BsAb could achieve localised activation of CD40 in tumours while simultaneously minimising the toxicity associated with systemic CD40 activation, thereby addressing the challenges encountered in current clinical practice. The finding highlights that both format of the molecule and affinity should be carefully chosen to balance required efficacy and conditional activation for CD40‒HER2 BsAbs. Indeed, CD40‒HER2 BsAb therapy potentiated the antitumour efficacy by remodelling macrophages and activating B cells, along with increasing the proportion of CD4+ and CD8+ T cells and decreasing the number of Tregs. In contrast, the therapy exhibited limited effects on DC in vivo, probably due to HER2 crosslinking impeding DC migration from tumours and inducing functional abnormalities (Figure 4F). Our data elucidate the mechanisms underlying the therapeutic efficacy of CD40‒HER2 BsAb, and future studies addressing the DC retention caused by TAA may contribute to the improvement of antitumour efficiency in vivo.

## AUTHOR CONTRIBUTIONS

Yuan Wang, Hongkai Zhang, Wei Wang and Yingbin Yan designed experiments and analysed the data. NaLi, Ruonan Li, Qiongqiong Ma, Xiaoqi Zhang, Wenxuan Ma, Yi Wang, Baoxin Duan, Kailu Yang, Dongping Zhang, Jiashuo Zhang, Manping Gu, Yaxing Wu, Jiajin Sun, Huawei Wang, Anqi Li and Yiyang Bai performed the experiments. Yuan Wang, Wei Wang and Na Li analysed the single‐cell sequencing data.Fuquan Chen, Yujie Tian and Xin Li offered some of the experiment resources and technical support. Yuan Wang, Hongkai Zhang and Na Li assisted in preparing the manuscript.

## CONFLICT OF INTEREST STATEMENT

The authors declare they have no conflicts of interest.

## ETHICS STATEMENT

All animal procedures complied with the Guide for the Care and Use of Laboratory Animals and were performed in accordance with the institutional ethical guidelines for animal experimentation. All experimental procedures were approved by the Research Ethics Committee of Nankai University.

## Supporting information



Supporting Information

Supporting Information

Supporting Information

Supporting Information

Supporting Information

Supporting Information

Supporting Information

Supporting Information

## Data Availability

The datasets analysed in this study are available from the corresponding author upon reasonable request.

## References

[1] Tang T , Cheng X , Truong B , Sun L , Yang X , Wang H . Molecular basis and therapeutic implications of CD40/CD40L immune checkpoint. Pharmacol Ther. 2021;219:107709. 33091428 10.1016/j.pharmthera.2020.107709 PMC7886970

[2] Vonderheide RH . CD40 agonist antibodies in cancer immunotherapy. Annu Rev Med. 2020;71:47‐58. 31412220 10.1146/annurev-med-062518-045435

[3] Knorr DA, Dahan R, Ravetch JV. Toxicity of an Fc‐engineered anti‐CD40 antibody is abrogated by intratumoral injection and results in durable antitumor immunity. Proc Natl Acad Sci U S A. 2018;115:11048‐11053. 30297432 10.1073/pnas.1810566115 PMC6205479

[4] White AL , Chan HTC , Roghanian A , et al. Interaction with FcγRIIB is critical for the agonistic activity of anti‐CD40 monoclonal antibody. J Immunol. 2011;187:1754‐1763. 21742972 10.4049/jimmunol.1101135

[5] Sum E , Rapp M , Dürr H , et al. The tumor‐targeted CD40 agonist CEA‐CD40 promotes T cell priming via a dual mode of action by increasing antigen delivery to dendritic cells and enhancing their activation. J Immunother Cancer. 2022;10:e003264. 35292514 10.1136/jitc-2021-003264 PMC8928381

[6] Ishihara J , Ishihara A , Potin L , et al. Improving efficacy and safety of agonistic anti‐CD40 antibody through extracellular matrix affinity. Mol Cancer Ther. 2018;17:2399‐2411. 30097487 10.1158/1535-7163.MCT-18-0091

[7] Traxlmayr MW , Lobner E , Antes B , et al. Directed evolution of Her2/neu‐binding IgG1‐Fc for improved stability and resistance to aggregation by using yeast surface display. Protein Eng Design Select. 2013;26:255‐265. 10.1093/protein/gzs102 23267121

[8] Orlova A , Magnusson M , Eriksson TLJ , et al. Tumor imaging using a picomolar affinity HER2 binding affibody molecule. Cancer Res. 2006;66:4339‐4348. 16618759 10.1158/0008-5472.CAN-05-3521

[9] Shields RL , Namenuk AK , Hong K , et al. High resolution mapping of the binding site on human IgG1 for Fc gamma RI, Fc gamma RII, Fc gamma RIII, and FcRn and design of IgG1 variants with improved binding to the Fc gamma R. J Biol Chem. 2001;276:6591‐6604. 11096108 10.1074/jbc.M009483200

[10] Lobner E , Humm A‐S , Göritzer K , et al. Fcab‐HER2 interaction: a Ménage à Trois. Lessons from X‐ray and solution studies. Structure. 2017;25:878‐889. 28528777 10.1016/j.str.2017.04.014

