# Supplementary material for: Bispecific antibody targeting CD40 and HER2 potentiates therapeutic efficacy by reprogramming macrophages within the tumour microenvironment
Source: Clin Transl Med. 2025 Jul 29;15(8):e70428. doi: 10.1002/ctm2.70428 (PMC12304727; doi:10.1002/ctm2.70428)
Supplement: Supplementary file 1 — Supporting Information [file CTM2-15-e70428-s003.docx]

**Supplementary information**

**• Materials and methods**

**• Supplemental figure legends**

**Materials and Methods**

**Cells**

Jurkat, human breast cancer cell lines BT474, SKBR3, T47D and MDA-MB-231 were obtained from the American Type Culture Collection (ATCC, USA). MB49 was obtained from Merk (Germany). MC38 was obtained from the Cell Bank of the Chinese Academy of Sciences (China). HEK293F and CHO cells were obtained from Thermo Fisher Scientific (USA). All cells were cultured in the recommended medium and incubated at 37 °C with 5% CO2. CHO-HER2 or CHO-CD40 cell line was generated by infection of CHO cells with human HER2 or CD40 expression lentivirus followed by fluorescence-activated cell sorting (FACS). MB49-hHER2 or MC38-hHER2 cell line was generated by infection of MB49 or MC38 cells with human HER2 expression lentivirus followed by FACS. The Jurkat/NF-κB-GFP-hCD40 reporter cell line was generated by infection of Jurkat cells with lentivirus expressing a GFP reporter under the control of 4 tandem repeats of the NFκB transcriptional response element for detection of NFκB pathway activity. The Jurkat/NF-κB-GFP cells were then infected with CD40-expressing lentivirus and stimulated with CD40L, subsequently followed by FACS.

**CD40-HER2 BsAbs construction**

CD40 binding scFv or Fab building blocks were constructed from a potent CD40 agonist antibody NK003 (C04) . ^1^ The HER2-binding scFv or Fab are derived from the clinically approved Trastuzumab (Herceptin®) or Pertuzumab (Perjeta®). Alternatively, the Fcab molecule STAB19 and the affibody molecule ZHER2 were used as building blocks of BsAb. The STAB19 was engineered by substituting amino acid sequences at the C-terminal structural loops in the CH3 domain of the human IgG1 Fc fragment to generate HER2 binding site. ^2^ The affibody ZHER2 was a small protein of 58 amino acids and bound to a conformational epitope on HER2 that was distant from those recognized by Trastuzumab. ^3,4^ Sequences of the VH and VL regions of Trastuzumab, Pertuzumab and APX005M were obtained from Drugbank and TABS Therapeutic Antibody Database. All required sequences were synthesized by Genewiz (China). The construction of CD40-HER2 BsAbs were performed according to standard recombinant DNA technologies. All CD40-HER2 BsAbs were cloned into the Pfuse expression vector and verified by sequencing.

**Production of CD40-HER2 BsAbs**

Antibody expression and purification were performed as previously reported. ^5^ The CD40-HER2 BsAbs expression vectors were transfected into HEK293F cells and cultured for 5 days. Supernatants were harvested and loaded onto Protein A resin (Genscript, China), eluted with glycine-HCl (pH 3.5) and neutralized with Tris-HCl (pH 8.0). Zeba Spin Desalting Columns (Thermo Fisher Scientific, USA) were used to exchange buffer of eluted protein to PBS. The BCA protein assay was used to quantify BsAbs.

**Jurkat/NF-κB-GFP-hCD40 reporter cell assays**

For the Jurkat/NF-κB-GFP-hCD40 reporter cell assays, 1.5 × 10^5^ breast cancer cells or CHO/CHO-HER2 cells were plated overnight at 37℃ in 48-well plates. Then, 1.5 × 10^5^ Jurkat/NF-κB-GFP-hCD40 reporter cells and different concentrations of CD40-HER2 BsAbs were added. We detected the GFP expression by using ﬂow cytometry. The results were analyzed using Flowjo X version 10.0.7 software. Subsequently, fluorescence intensity was calculated as the percentage of GFP-positive cells multiplied by the mean fluorescence intensity (MFI). The resulting data were then plotted against the antibody concentrations and mapped using GraphPad Prism software.

**Antigen-binding capacity assays**

The binding of BsAbs to CD40 or HER2 protein was measured by surface plasmon resonance (SPR), flow cytometry analysis and confocal microscopy assays. For affinity assay, we performed on a Biacore T200 at 25 °C using running buffer HBS-EP (recipe). The antigen CD40 or HER2 was immobilized directly onto a CM5 sensor chip, different concentrations of BsAbs were loaded at 30 μL/min for 120 s, then dissociated at 30 μL/min for 240 s. For the epitope binding experiment, antigen CD40 was coupled to a CM5 sensor chip, 0.5 μM CD40 mAb-H and APX005M were sequentially loaded for 120 s followed by dissociation for 80 s. The sensor chip was regenerated with glycine (recipe) at 30 μL/min for 30 s. Results were simulated and calculated using BIAevaluation software T200. The binding of CD40-HER2 BsAb-11 to cell surface human CD40 and HER2 was assessed by flow cytometry analysis and confocal microscopy. BsAb was fluorescently labelled with sulfo-cyanine-7-succinimidyl ester (MCE, USA) according to the manufacturer's instructions, CD40-expressing Jurkat cells or HER2-expressing CHO cells were incubated with serially diluted Cy7-labelled BsAb at 4°C for 30 minutes. Then, we collected the cells and measured Cy7 fluorescence using flow cytometry, EC50 was calculated using GraphPad Prism. For dual binding capacity assay, CHO-CD40 and CHO-HER2 cells were labelled with PKH26 (Sigma-Aldrich, USA) and PKH67 (Sigma-Aldrich, USA) according to the manufacturer's instructions, respectively. The labelled CHO-CD40 and CHO-HER2 cells were incubated with antibodies at 4°C for 30 minutes. The samples were measured using flow cytometry and visualized by confocal microscopy (Leica TCS SP8).

**In vitro APCs activation assays**

For DC activation, we performed BMDC induction and fluorescent bead phagocytosis experiments. Briefly, bone marrow cells isolated from hCD40tg mice were induced with GM-CSF (100 ng/mL) and IL-4 (50 ng/mL) for 7 days and stimulated with LPS (100 ng/mL) to generate BMDCs. Next, the yellow-green NeutrAvidin™-labelled microspheres were incubated with biotinylated human HER2 for 1 h at room temperature. Then, HER2-coated microspheres were incubated with antibodies for 2 h at 37°C. Subsequently, the fluorescent microsphere-HER2-antibody complex was added to BMDCs and cultured at 37°C for 3 hours. The phagocytosis of BMDCs was observed by confocal microscopy and analyzed by using flow cytometry.

For B cell activation, we isolated B cells from human PBMCs by using CD19 beads (Miltenyi, Germany). Then, 8 × 10^4^ SKBR3 cells were plated in 96-well overnight at 37℃. Thereafter, 1 × 10^5^ B cells were added, along with different concentrations of BsAbs, and the co-cultures were maintained for 48 h. The activation marker CD86 was analyzed by flow cytometry.

For macrophages activation, we isolated peritoneal macrophages from hCD40tg mice and co-cultured them with MC38-HER2-OVA at a ratio of 3:1 in 48-well plates. The plates were then incubated for 24 h in the presence or absence of antibodies. The expression of OVA and CD86 on the surface of F4/80 cells was detected by flow cytometry.

**In vivo anti-tumor activity assays**

Six to eight-week-old hCD40tg mice were purchased from the Shanghai Model Organisms Center (China), and all mice were housed under SPF conditions. The hCD40tg mice were injected subcutaneously with MB49-hHER2 or MC38-hHER2 cells. When tumor volumes reached 70 to 100 mm^3^, mice were randomly assigned to different groups and treated intraperitoneally with different antibodies every three days. Blood, liver and tumor were collected 96 hours after the last antibody treatment. Tumor volume (V) was monitored every three days by measuring the length (L) and width (W) of the tumors using electronic calipers and calculated using the formula (L × W ^2^) × 0.5. For antibody toxicity analysis, AST and ALT were detected in mouse blood samples using the AST and ALT ELISA kits, and tissue injury was further assessed by histopathological analysis with HE staining in liver and kidney sections. For survival analysis, mice died when the tumor volume reached 2000 mm^3^, and survival curves were plotted using Graph Pad based on mouse death time. For flow cytometric detection of immune cells, liver or tumor single cell suspensions were prepared and depleted of erythrocytes. Then the cells were resuspended in 100 µl PBS with 2% FBS buffer containing the staining antibody and analyzed by flow cytometry on a BD Fortessa. For immune cell depletion experiments, MB49-hHER2 tumor-bearing hCD40tg mice were treated by intraperitoneal injections of anti-CD3, anti-CD19, anti-CSF1R, anti-CD4, anti-CD8, and anti-CD25 monoclonal antibodies, respectively, 24 hours before CD40-HER2 BsAb therapy. Plot survival curves using Graph Pad based on time of death of mice.

**ScRNA-seq of tumor-infiltrating immune cells**

For single-cell sequencing analysis, tumor-bearing hCD40 mice were euthanized 72 hours after three rounds of antibody treatment. Single cell sequencing was performed as previously described. ^6^ Briefly, tumor tissues were digested into single-cell suspensions and dead cells were removed, followed by separation of immune cells with CD45 beads. The collected CD45-positive cells were encapsulated and sequenced using Chromium Next GEM Single Cell 3ʹ Reagent Kits v3.1 (10x Genomics). The raw sequencing data were analyzed using Cell Ranger software. Gene barcode matrices generated by Cell Ranger (version 4.1.1) pipeline for quality control and subsequent analysis. ^7^ Low-quality cells (< 100 genes/cell, > 7,000 genes/cell, > 50,000 UMIs/cell, > 20% mitochondrial) were filtered out. The data were then normalized, and highly variable genes were detected using the FindVariableFeatures function. To reduce the dimensionality of the scRNA-Seq dataset, principal component analysis was performed. Batch correction was conducted using the Harmony package (version 1.0). ^8^ Cell clusters were identified with the FindClusters function in Seurat at a resolution of 0.8. Each cluster was manually classified into known biological cell types. These clusters were visualized using the uniform manifold approximation and projection (UMAP) plots. T cells and NK cells, as well as macrophages, were re-clustered and annotated into specific cell types. Use the FindMarkers function in Seurat to identify cell cluster specific genes or differentially expressed genes in individual cell clusters. Calculate the average expression of genes within each cell cluster. Use WebGestalt for Gene Set Enrichment Analysis (GSEA) to identify enriched pathways annotated in the GO database .^9,10^ Limit the pathway size to 500 genes per pathway. Rank genes based on log2 fold change calculated by the FindMarkers function in Seurat. Use 1,000 permutations to estimate the FDR for GSEA analysis. Use the ComplexHeatmap package (version 2.8.0) in R to generate a heatmap of the average gene expression or the pathway normalized enrichment score generated by GSEA ^11^.

**Statistical analysis**

Data were analyzed with GraphPad Prism. Statistical significance was assessed by comparing mean values (± SD) using the student’s *t* test for independent groups as follow: *, *P <* 0.05; **, *P <* 0.01; ***, *P <* 0.001; ****, *P <* 0.0001.

**REFERENCES**

1. Wang Y, Jin R, Shen B, Li N, Zhou H, Wang W, et al. High-throughput functional screening for next-generation cancer immunotherapy using droplet-based microfluidics. Sci Adv. 2021; 7: eabe3839.
2. Traxlmayr MW, Lobner E, Antes B, Kainer M, Wiederkum S, Hasenhindl C, et al. Directed evolution of Her2/neu-binding IgG1-Fc for improved stability and resistance to aggregation by using yeast surface display. Protein Engineering, Design and Selection. 2013; 26: 255-65.
3. Orlova A, Magnusson M, Eriksson TLJ, Nilsson M, Larsson B, Höidén-Guthenberg I, et al. Tumor Imaging Using a Picomolar Affinity HER2 Binding Affibody Molecule. Cancer Research. 2006; 66: 4339-48.
4. Eigenbrot C, Ultsch M, Dubnovitsky A, Abrahmsén L, Härd T. Structural basis for high-affinity HER2 receptor binding by an engineered protein. Proc Natl Acad Sci USA. 2010; 107: 15039-44.
5. Yea K, Zhang H, Xie J, Jones TM, Lin C-W, Francesconi W, et al. Agonist antibody that induces human malignant cells to kill one another. Proc Natl Acad Sci U S A. 2015; 112: E6158-65.
6. Liu S, Li F, Ma Q, Du M, Wang H, Zhu Y, et al. OX40L-Armed Oncolytic Virus Boosts T-cell Response and Remodels Tumor Microenvironment for Pancreatic Cancer Treatment. Theranostics. 2023; 13: 4016-29.
7. Hao Y, Hao S, Andersen-Nissen E, Mauck WM, Zheng S, Butler A, et al. Integrated analysis of multimodal single-cell data. Cell. 2021; 184: 3573-3587.
8. Korsunsky I, Millard N, Fan J, Slowikowski K, Zhang F, Wei K, et al. Fast, sensitive and accurate integration of single-cell data with Harmony. Nat Methods. 2019; 16: 1289-96.
9. Liao Y, Wang J, Jaehnig EJ, Shi Z, Zhang B. WebGestalt 2019: gene set analysis toolkit with revamped UIs and APIs. Nucleic Acids Research. 2019; 47: W199-205.
10. Subramanian A, Tamayo P, Mootha VK, Mukherjee S, Ebert BL, Gillette MA, et al. Gene set enrichment analysis: A knowledge-based approach for interpreting genome-wide expression profiles. Proc Natl Acad Sci USA. 2005; 102: 15545-50.
11. Gu Z, Eils R, Schlesner M. Complex heatmaps reveal patterns and correlations in multidimensional genomic data. Bioinformatics. 2016; 32: 2847-9.

**SUPPLEMENTAL Figure Legends**

**Figure S1 Generation and optimization of CD40-HER2 BsAbs.** (A) CD40 activation in reporter cells stimulated by CD40 mAb-H with or without anti-Fc antibody. (B) The binding of CD40-HER2 BsAbs to both CD40 (left) and HER2 (right) was assessed by surface plasmon resonance. (C) The binding of CD40 mAb-H and APX005M to different epitopes of CD40 was determined by surface plasmon resonance. (D) Schematic diagram of CD40-HER2 BsAbs with different epitopes. (E) The binding of CD40-HER2 BsAbs with different epitopes to both CD40 (up) and HER2 (down) was assessed by surface plasmon resonance. (F) CD40 activation in reporter cells stimulated by different formats of BsAbs with different epitopes in the presence of CHO-HER2 or CHO cells. (G) The cell surface expression of HER2 on different breast cancer cell lines was analyzed using flow cytometry. (H) CD40 activation in reporter cells stimulated by different formats of BsAbs in the presence of breast cancer cell lines. **p* < 0.05, ***p* < 0.01, ****p* < 0.001, *****p* < 0.0001.

**Figure S2 In vitro functionality and in vivo activity of CD40-HER2 BsAbs.** (A) The affinity of CD40-HER2 BsAb-11 for CD40 and HER2 was measured by surface plasmon resonance. (B) The binding ability of CD40-HER2 BsAb-11 to cell surface CD40 (Jurkat/NF-κB-GFP-hCD40) and HER2 (CHO-HER2) was determined by flow cytometry. (C) The simultaneous binding capacity of CD40-HER2 BsAb-11 to HER2-expressing (green) and CD40-expressing (red) cells was measured by flow cytometry. (D) Phagocytosis efficiency of HER2+ beads by BMDC in the presence or absence of CD40-HER2 BsAb-11 or CD40 mAb-H-mIgG1 in Figure 2B was detected by flow cytometry. (E, F) Survival and tumor growth in MC38-hHER2 (E) and MB49-hHER2 (F) tumor-bearing hCD40tg mice treated as indicated (n=5). (G) Hematoxylin-eosin (HE) staining of liver and kidney tissues from MB49-hHER2 bearing hCD40tg mice 72 hours after indicated treatment. **p* < 0.05, ***p* < 0.01, ****p* < 0.001, *****p* < 0.0001.

**Figure S3 Immune cell gating strategy in MB49-hHER2 mouse model.** A step-by-step gating strategy for different immune cell subpopulations.

**Figure S4 Analysis of tumor-infiltrating immune cells by scRNA-seq in MB49-hHER2 tumor.** (A) Bubble plots showed the expression of cell markers in each cluster. (B) Heatmap of gene expression of different signaling pathways between B cell subtypes. (C) Bubble plots showed the expression of cell markers in macrophage clusters. (D) Distribution of different macrophage clusters. (E) Heatmap of gene expression of different signaling pathways between macrophage subtypes. (F) Bubble plots showed the expression of cell markers in T cell clusters.

**Figure S5 The individual tumor growth curve of mice depleted different immune cells.** (A, B) MB49-hHER2 bearing hCD40tg mice was treated with different immune cell depletion antibodies 24 h before the injection of CD40-HER2 BsAb-11. (A) The individual tumor growth of mice depleted macrophages, B cells, T cells was monitored every 3 days (n=6). (B) The individual tumor growth of mice depleted CD4+, CD8+ T cells, Tregs was monitored every 3 days (n=5).
