# Supplementary figures and images for: Bispecific antibody targeting CD40 and HER2 potentiates therapeutic efficacy by reprogramming macrophages within the tumour microenvironment
Source: Clin Transl Med. 2025 Jul 29;15(8):e70428. doi: 10.1002/ctm2.70428 (PMC12304727; doi:10.1002/ctm2.70428)

Figure S1

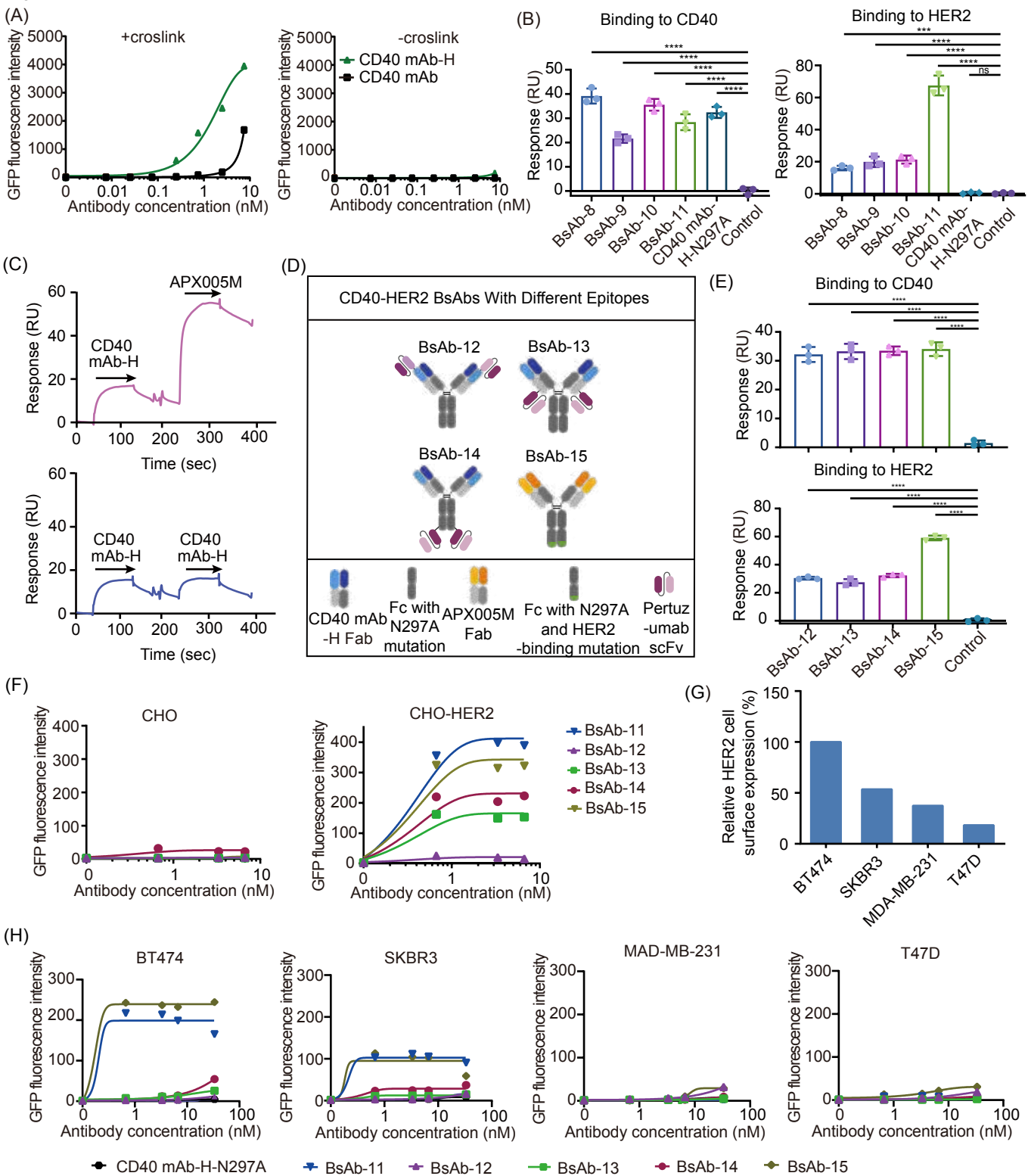

Supplement: Supplementary file 2 — Supporting Information [file CTM2-15-e70428-s007.pdf]

Figure S2

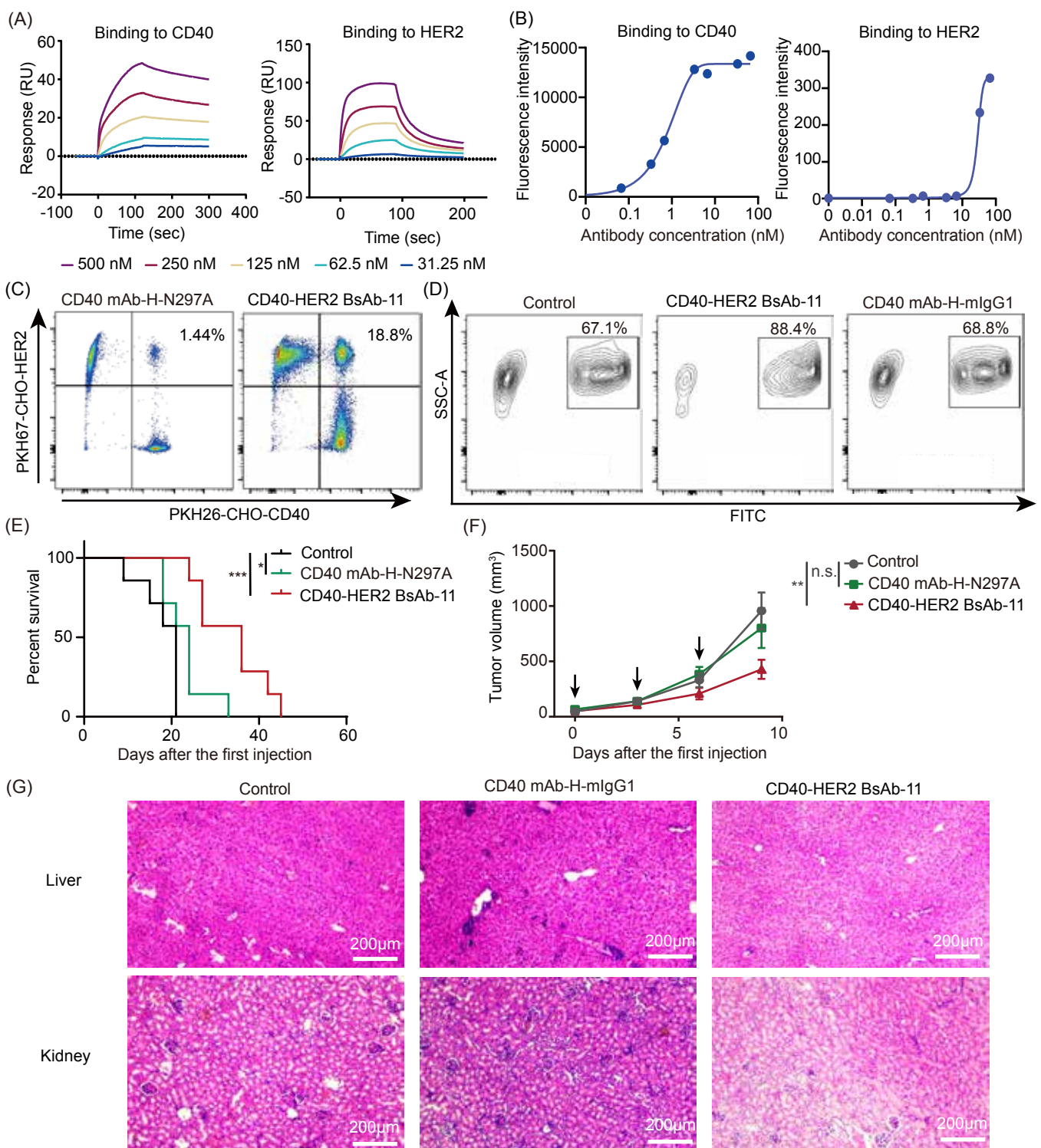

Supplement: Supplementary file 3 — Supporting Information [file CTM2-15-e70428-s004.pdf]

Figure S3

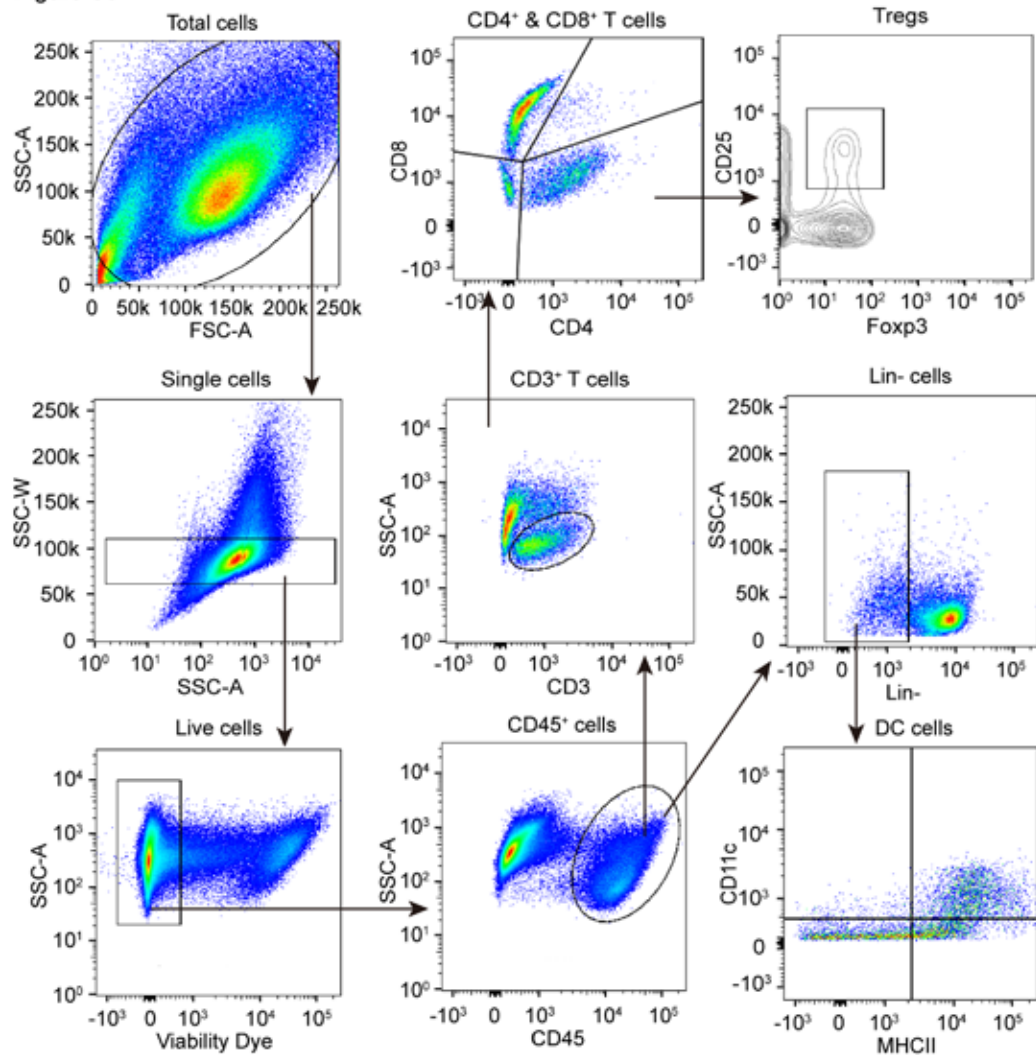

Supplement: Supplementary file 4 — Supporting Information [file CTM2-15-e70428-s001.pdf]

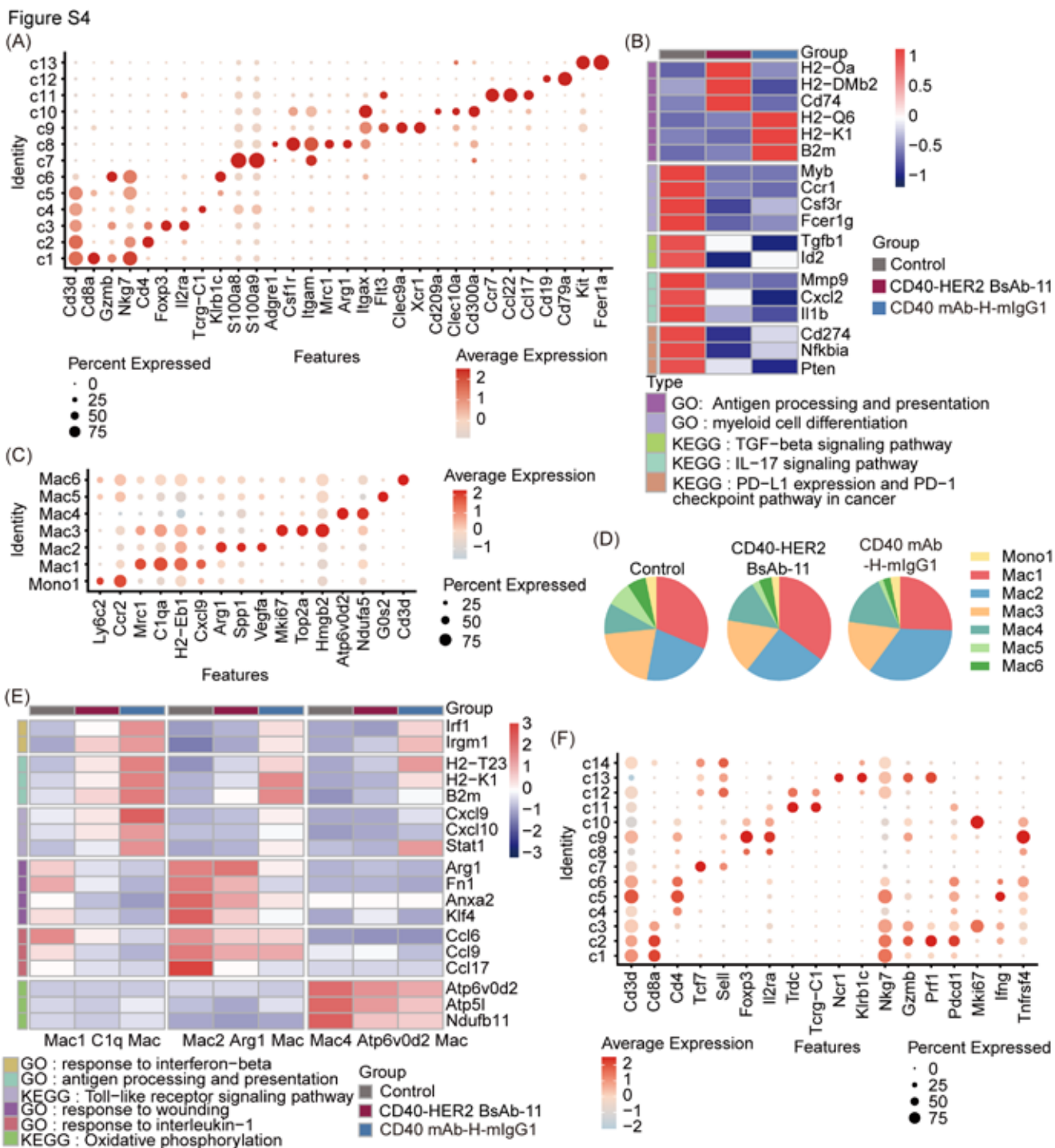

Supplement: Supplementary file 5 — Supporting Information [file CTM2-15-e70428-s002.pdf]

Figure S5

(A)

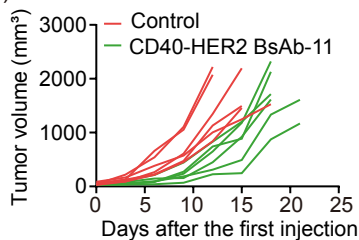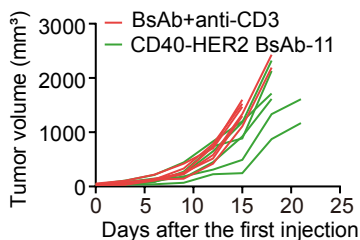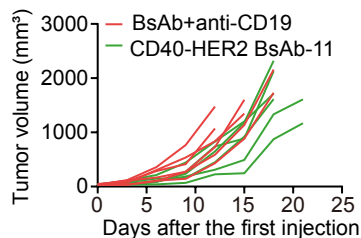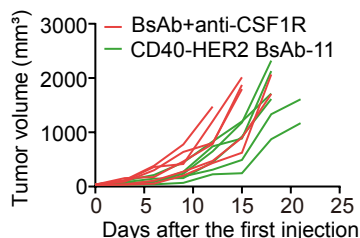

(B)

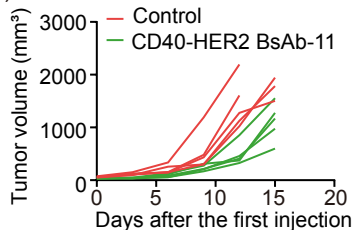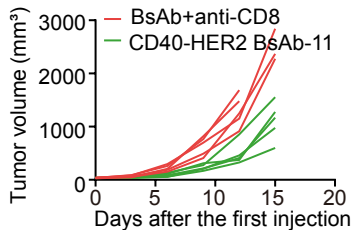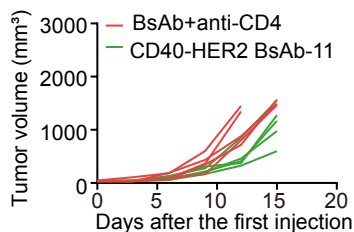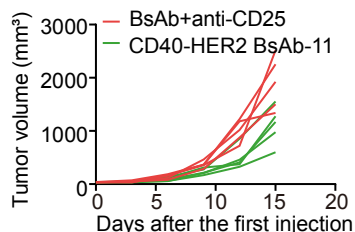

Supplement: Supplementary file 6 — Supporting Information [file CTM2-15-e70428-s006.pdf]
